# Supplementary material for: Dual-Task Performance, Balance and Aerobic Capacity as Predictors of Falls in Older Adults with Cardiovascular Disease: A Comparative Study
Source: Behav Sci (Basel). 2023 Jun 9;13(6):488. doi: 10.3390/bs13060488 (PMC10295188; doi:10.3390/bs13060488)
Supplement: Supplementary file 1 [file behavsci-13-00488-s001.zip › behavsci-2390659-supplementary.pdf]

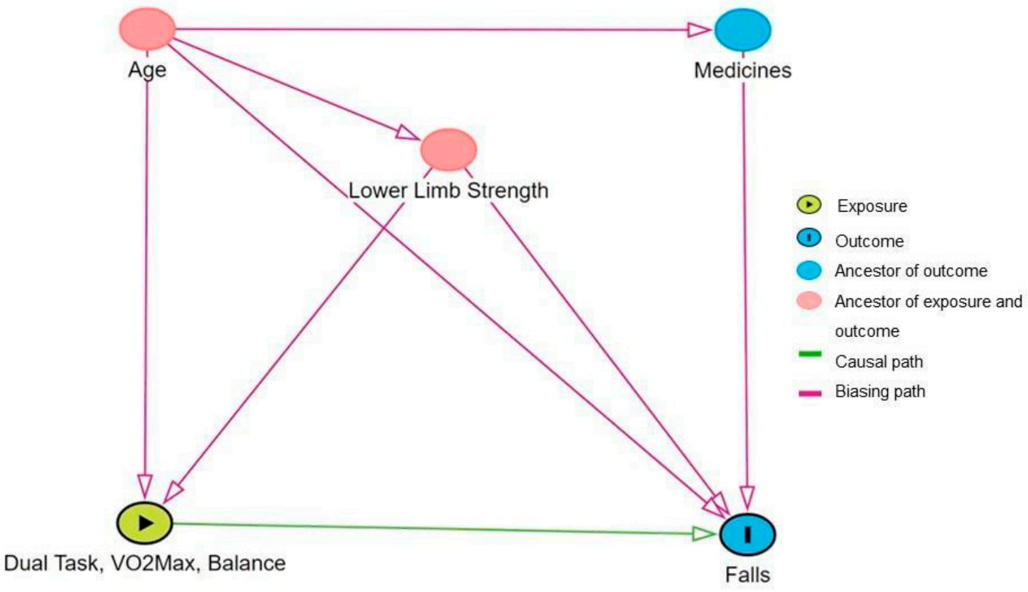

Figure S1. Causal diagram (DAG) constructed to guide the selection of the possible adjustment variables.
